# Detection of *Vibrio vulnificus* in Seafood With a DNAzyme-Based Biosensor

**DOI:** 10.3389/fmicb.2021.655845

**Published:** 2021-06-04

**Authors:** Shihui Fan, Chao Ma, Xiaopeng Tian, Xiaoyi Ma, Mingcan Qin, Hangjie Wu, Xueqing Tian, Jing Lu, Mingsheng Lyu, Shujun Wang

**Affiliations:** ^1^Jiangsu Key Laboratory of Marine Bioresources and Environment/Jiangsu Key Laboratory of Marine Biotechnology, Jiangsu Ocean University, Lianyungang, China; ^2^Co-Innovation Center of Jiangsu Marine Bio-Industry Technology, Jiangsu Ocean University, Lianyungang, China; ^3^Jiangsu Marine Resources Development Research Institute, Lianyungang, China

**Keywords:** *Vibrio vulnificus*, DNAzyme, rapid detection, fluorescence sensor, aquatic products

## Abstract

*Vibrio vulnificus* is an important pathogenic bacterium that is often associated with seafood-borne illnesses. Therefore, to detect this pathogen in aquatic products, a DNAzyme-based fluorescent sensor was developed for the *in vitro* detection of *V. vulnificus*. After screening and mutation, a DNAzyme that we denominated “RFD-VV-M2” exhibited the highest activity, specificity, and sensitivity. The limit of detection was 2.2 × 10^3^ CFU/ml, and results could be obtained within 5–10 min. Our findings suggested that the target of DNAzyme RFD-VV-M2 was a protein with a molecular weight between 50 and 100 kDa. The proposed biosensor exhibited an excellent capacity to detect marine products contaminated with *V. vulnificus*. Therefore, our study established a rapid, simple, sensitive, and highly specific detection method for *V. vulnificus* in aquatic products.

## Introduction

Currently, bacterial infections have become a serious global problem. It is also a significant challenge for people to detect specific bacteria from marine biological samples. Besides, microbiological techniques, although highly accurate, need several days to obtain a result (Ali et al., 2011). Therefore, it is necessary to develop a simple molecular probe, which is highly specific for pathogenic strains of bacteria (Shen et al., 2015).

DNAzymes often refer to single-stranded DNA molecules with catalytic capabilities (Breaker, 1997). RNA-cleaving DNAzymes usually exist in random-sequence synthetic DNA libraries and can be isolated using *in vitro* selection (Ali et al., 2011). These DNAzymes have a specific ability to cleave a DNA/RNA substrate at the location of a designated ribonucleotide and exhibit excellent stability, catalytic efficiency, specificity, affinity, and sensitivity, which makes them uniquely suited for a number of applications including biosensors and pathogen detection (Ali et al., 2019; Gu et al., 2019). The first DNAzyme for RNA cleavage was reported in 1994 using an *in vitro* selection designed with metal ions (Breaker and Joyce, 1994). Since then, many DNAzymes have been selected and applied to detect metals such as Zn^2+^, Cu^2+^, UO^2+^, Pb^2+^, Ag^+^, and Hg^2+^ (Liu and Lu, 2007; Luo et al., 2012; Endo et al., 2015; Saran and Liu, 2016; Yun et al., 2016). Besides, in recent years, many RNA-cleaving fluorogenic DNAzymes (RFDs) have been selected through crude extracellular mixtures (CEM) and detect common bacteria such as *Escherichia coli*, *Klebsiella Pneumoniae*, *Helicobacter pylori*, *Bacterial pathogen*, and *Legionella pneumophila* (Zhang et al., 2016). Considering these successful applications, it is possible to derive a novel RNA-cleaving DNAzyme as an effective molecular recognition element for the simple detection of pathogens (Ma et al., 2021; Rothenbroker et al., 2021).

*Vibrio vulnificus* is a Gram-negative and halophilic bacterium that has been largely associated with seafood-borne diseases, which can be lethal in some circumstances (Strom and Paranjpye, 2000; Gulig et al., 2005). *V. vulnificus* contamination can occur in a variety of seafood including shrimp, fish, oysters, and crab, as well as raw or undercooked seafood (Paydar and Thong, 2013; Phillips and Satchell, 2017; Bonnin-Jusserand et al., 2019). The main symptoms of patients infected with *V. vulnificus* include gastroenteritis, primary traumatic infection, and sepsis. *V. vulnificus* primary septicemia has a mortality rate of up to 60%, which highlights the serious public health and food safety concerns posed by this pathogen (Pei et al., 2017). Therefore, specific and reliable detection methods for *V. vulnificus* that can be performed quickly and on-site are critical to better control its spread.

There are several methods to detect *V. vulnificus*. The conventional method is to enrich samples in a specific medium, after which phenotypic or physiological and biochemical characteristics are collected. Polymerase chain reaction (PCR) and real-time PCR-based approaches have also been developed for the detection of *V. vulnificus* (Campbell and Wright, 2003; Panicker et al., 2004; Çam et al., 2019). Loop-mediated isothermal amplification (LAMP) technology is another sensitive method (Ren et al., 2009; Srividya et al., 2019). However, the aforementioned approaches are time-consuming, labor-intensive, and require highly specialized and often costly equipment. Therefore, given the pressing need for innovative methods for *V. vulnificus* detection, our study sought to develop a sensitive, convenient, and rapid *V. vulnificus* biosensor.

Specifically, DNAzymes were selected from available libraries, from which a DNAzyme-based biosensor was selected to detect *V. vulnificus* in aquatic products. The selected biosensor exhibited good specificity and excellent sensitivity, with a limit of detection of 2.2 × 10^3^ CFU/ml. Moreover, this novel biosensor enabled convenient on-site detection of *V. vulnificus* and can be stored at room temperature for over 6 months. Therefore, the proposed DNAzyme-based biosensor is uniquely well suited for the rapid detection of *V. vulnificus* in the seafood industry.

## Materials and Methods

### Chemicals and Bacterial Strains

DNA sequences for *in vitro* selection and streptavidin-coated magnetic particles were purchased from Sangon Biotech (Shanghai, China) (Table 1). *Vibrio vulnificus*, *Vibrio anguillarum*, *Pseudomonas aeruginosa*, and *Edwardsiella tarda* were obtained from the China Center of Industrial Culture Collection (CICC). *Vibrio alginolyticus*, *E. coli*, *Bacillus subtilis*, and *Staphylococcus aureus* were provided by the Marine Resources Development Institute of Jiangsu (Lianyungang, China). Tryptone and yeast extract powder were purchased from Oxoid (Basingstoke, England). Agarose was purchased from Solarbio Life Science (Beijing, China). Tris (hydroxymethyl)-aminomethane (Tris), Tween-20, and metal salts were acquired from Sinopharm (Beijing, China), all of which were of the highest purity available. N-(2-Hydroxyethyl) piperazine-N′-(2- ethane-sulfonic acid) (HEPES), 2-(N-morpholino) ethane sulfonic acid (MES), deoxynucleotide (dNTP) mix, Taq DNA polymerase with 10× PCR buffer, 6× gel loading dye, and a low molecular weight DNA ladder were purchased from New England Biolabs (Beijing, China). Pullulan, 40% acryl/bis solution (29:1), and Gel-red (10,000×) were obtained from BBI Co., Ltd. (Shanghai, China).

**TABLE 1:** DNA sequences related to *in vitro* selection.

**No.**	**DNA names**	**Sequences and modifications (5′-3′)**
1	Lib-3-N35-pool	**Phosp**-GGAAGCAGCCCCCACTGCTT-N35-TTCTGTTGACGACCACGATT
2	Lib-3-P1	**Biotin**- GTCAACAGAATrAGGAAGC AGCCCCCA
3	Lib-3-P2	AATCGTGGTCGTCAACAGAA
4	FAM-substrate	**FAM**-GTCAACAGAATrAGGAAGCAGCCCCCA
5	L-vvh	TTCCAACTTCAAACCGAACTATGA
6	R-vvh	ATTCCAGTCGATGCGAATACGTTG

***Phosp** was used for Taq DNA Polymerase to build library, **Biotin** was used for in vitro screening, and **FAM** was used for fluorescence detection.*

### *Vibrio vulnificus* Crude Extracellular Mixture (CEM) Preparation

The lyophilized *V. vulnificus* strain was revitalized in 20 ml of Luria Bertani (LB) medium (1% tryptone, 0.5% yeast extract, and 1% NaCl, pH 7.5) and incubated at 30°C with 180 rpm until it reached an approximate OD_600_ (optical density at 600 nm) of 0.8. The broth was then separated into several 1.5-ml sterilized microcentrifuge tubes and centrifuged at 12,000 rpm for 5 min at 4°C. The supernatant was stored at −20°C as the CEM of *V. vulnificus* (CEM-VV) for downstream experiments. CEMs of other bacteria for the detection of specificity were prepared according to their optimal culture conditions.

### *In vitro* Selection

For the purification of the library, the Lib-3-N35-Pool Library contains 35 random sequences of bases as the DNAzyme library, whereas Lib-3-P1 contains biotin-labeled and adenine oligonucleotide (rA) sequences (Table 1). First, biotin-bearing and rA sequences were connected to the Lib-3-N35-Pool Library using PCR [DNA Library (20–100 ng/μl)]. To achieve this, 1 μl of Lib-3-N35-Pool Library, 2 μl of Lib-3-P1 and Lib-3-P2 (10 mol/μl each), 8 μl of 10× PCR buffer (with MgCl_2_), 1.5 μl of dNTP mixture (10 μM), 1 μl of Taq DNA polymerase (1.25 U/μl), and 34.5 μl of ddH_2_O were added to PCR tubes. The PCR cycling parameters were as follows: 94°C for 5 min; 94°C for 30 s, 55°C for 30 s, and extension at 72°C for 1 min, 20 cycles; final extension at 72°C for 2 min. PCR products were purified via 10% denatured polyacrylamide gel electrophoresis (dPAGE) and used as DNA templates (dilute to 100 ng/μl) for subsequent experiments.

**FIGURE 1:**
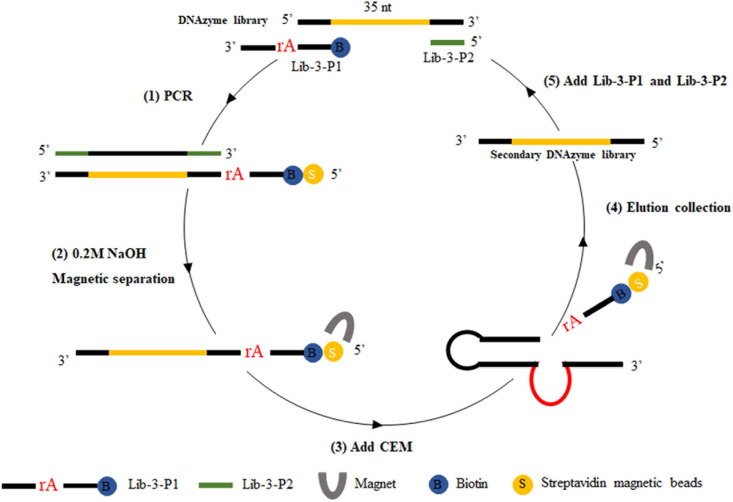
CEM-VV-specific DNAzyme selection. Briefly, (1) biotin-containing tags were fixed to the original DNAzyme library by polymerase chain reaction (PCR), which could be attached to the streptavidin-coated magnetic beads. (2) The magnetic beads were washed with 0.2 M NaOH to remove the DNA chain, which was not connected. (3) DNA was bound to crude extracellular mixtures of *Vibrio vulnificus* (CEM-VV) to form a specific structure, resulting in a cleavage reaction. (4) Magnetic separation and alcohol precipitation were used to recover DNA as a library of next round selection. (5) Lib-3-P1 and Lib-3-P2 were added for polymerase chain reaction (PCR), and the next round of selection was performed.

For positive selection, the libraries required for each screening round were prepared by the magnetic bead-PCR method, as illustrated in Figure 1. In each round of screening, 50 μl of streptavidin-coated beads were washed three times using buffer A (10 mM Tris–HCl, 1 mM EDTA, 1 M NaCl, 0.02%, Tween 20, pH 7.5). Afterward, the magnetic beads were incubated with biotin-labeled DNA (500 μl) at 37°C for 30 min. The unbound DNA (supernatant) was removed by magnetic separator. The supernatant was discarded and washed twice with 500 μl of avidin reaction buffer. The magnetic beads were then washed with 0.2 M NaOH, and the supernatant was discarded to remove the DNA chain without connection. The magnetic beads were then washed again with ultrapure water to ensure that the pH value remained at 7.0. Then, 150 μl of CEM-VV was mixed evenly with an equal volume of buffer B (100 mM HEPES, 300 mM NaCl, 30 mM MgCl_2_, 0.02% Tween 20, pH 7.5) to the magnetic beads for 1 h at 37°C for cleavage reaction. Finally, the cleavage DNA fragments obtained from the reaction were recovered through alcohol precipitation after magnetic separation as the library of the next round selection. The optimal cycle number was selected for PCR amplification, and the PCR products were recovered by alcohol precipitation to carry out the next cycle.

### Activity Assay

**FIGURE 2:**
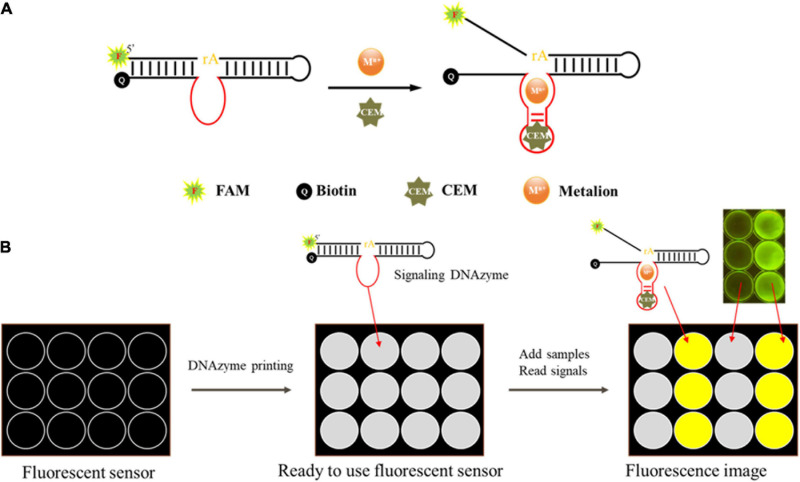
Schematic of the mode of action of the DNAzyme and the envisioned DNAzyme sensor. **(A)** The DNAzyme becomes activated upon interaction with the bacteria-associated targets. The active DNAzyme then cleaves the fluorogenic substrate to produce a fluorescent signal. **(B)** A sensor was made to detect fluorescence. The DNAzyme sensor mixed with pullulan + trehalose + DNAzyme was taken into each well and air dried. Samples were then applied to the test zones and allowed to react. The DNAzyme cleaved the fluorogenic substrate and produced a fluorescent signal, which could be monitored using a fluorescent scanner and analyzed using specialized software when a sample contained the target bacteria.

DNAzyme candidates were synthesized by Sangon Biotech. The substrate labeled with carboxyfluorescein (FAM) at the 3′ end and the DNAzyme labeled with a quencher at the 5′ end were complexed in buffer B (100 mM HEPES, 300 mM NaCl, 30 mM MgCl_2_ 0.02% Tween 20, pH 7.5) and annealed at 95°C for 1 min. The mixture was then allowed to stay at room temperature to form the DNAzyme complex (final concentration of 5 μM). For activity detection, 20 μl of buffer B, 15 μl of ultrapure water, 5 μl of DNAzyme complex (5 μM), and 10 μl of CEM-VV were mixed for 60 min at room temperature (Figure 2A). Gel dye (with 8 M urea) was then added to terminate the reaction. The products were then separated via 15% dPAGE at 150 V for 80 min, and gel bands were analyzed with a Bio-Rad Gel Doc^TM^ EZ imaging system.

### Screening for Active DNAzyme

#### Enzyme Assays

DNAzyme activity was detected using the fluorescence method in 96-well plates. In each well, 45 μl of buffer B, 41 μl of ultrapure water, and 4 μl of DNAzyme complex were mixed, after which 10 μl of CEM-VV was added. Fluorescence (F) was monitored with a micro board reader (Infinite M1000 Pro, Tecan, Switzerland; excitation wavelength = 488 nm, emission wavelength = 520 nm). Broth was added instead of CEM-VV to serve as a control (F_0_). The DNAzyme activity was dependent on F − F_0_. Fluorimetry was used for sensitivity analysis, and three independent test results were analyzed. The error bars represent standard deviations, and SPSS software was used to analyze significant difference. The signal dynamics were monitored and tracked on a microplate reader at 30-s intervals for 1 h.

### Optimization of Detection Conditions

Next, we selected an optimal buffer solution pH from 4.5 to 8.0. Buffer B was prepared using 100 mM MES (pH 4.5, 5, 5.5, 6, 6.5) and 100 mM HEPES, (pH 7, 7.5, 8). Buffer B was also added with 300 mM NaCl, 30 mM MgCl_2_, and 0.02% Tween-20.

To select optimal bivalent metal ion concentrations, different divalent metal ions (Mg^2+^, Ca^2+^, Sr^2+^, Ba^2+^, Mn^2+^, Co^2+^, Fe^2+^, and Ni^2+^) with 30 mM were incorporated into buffer B. Here, different concentrations of Mg^2+^ (0, 1, 2, 5, 10, 15, 20, 25, 30, 60, 90, 120, 150, 180, 210, 240, 270, and 300 mM) were prepared. The reactions were then performed under optimal pH conditions.

### Specificity and Sensitivity of Truncated DNAzyme

Different bacteria (*V. alginolyticus*, *V. anguillarum*, *P. aeruginosa*, *E. tarda*, *E. coli*, *B. subtilis*, and *S. aureus*) were cultured under their optimal culture conditions. All of the bacteria were centrifugated with 12,000 rpm for 5 min, and the supernatant was taken as the CEM. The detections were performed under optimal conditions of DNAzyme detection, as described above. The results were detected using fluorescence intensity and 15% dPAGE analyses.

RFD-VV-M2 exhibited the highest activity among all truncated DNAzymes analyzed herein. The sensitivity of DNAzyme RFD-VV-M2 was then evaluated as described by a previous study (Aguirre et al., 2013). *V. vulnificus* was cultivated in LB solid medium to OD_600_ around 0.8, then 10-fold serial dilution for eight gradients, and taken 100 μL of each diluent spread on LB solid medium. The plates were incubated at 30°C for 12 h, and the colony-forming units (CFU) were counted. Then, *V. vulnificus* was centrifuged at 12,000 rpm for 5 min at 4°C, and the supernatant was taken as CEM-VV. The DNAzyme reaction was then monitored by measuring fluorescence changes for 1 h and analyzed via 15% dPAGE.

### DNAzyme RFD-VV-M2 Optimization

Different concentrations of DNAzyme RFD-VV-M2 (100, 200, and 300 nM) were incorporated into a mixture of 10 μl of 8% pullulan (PL), 10 μl of 0.25 M trehalose (TH), and 15 μl of 2× SB solution. The solution was then fixed onto a polystyrene board to detect fluorescence when CEM-VV was added. Similarly, the reaction time was set from 0 to 20 min, and the fluorescence could be observed with a Blue Light Gel Imager instrument.

### Protein-Dependent DNAzyme RFD-VV-M2 Selectivity

We next investigated the target of DNAzyme RFD-VV-M2. CEM-VV was first digested using trypsin and protease K. Then, the CEM-VV was filtered through different molecular weights ranging from 10 to 100 kDa. The results were then analyzed via 15% dPAGE.

### Design of DNAzyme-Based Sensor

We designed a fluorescent sensor based on DNAzyme to detect *V. vulnificus* using a polypropylene board (Ali et al., 2017), and the details are described in Figure 2B. In this sensor, 5 μl of DNAzyme, 0.25 M trehalose (TH), and 8% pullulan (PL) were mixed evenly, and the mixture was dripped onto the board. Then, the board was dried at 60°C and stored at room temperature before use. Upon detection, 30 μl of the template (CEM-VV) was added onto the sensor, and the fluorescence was detected with a Blue Light Gel Imager instrument after 10 min.

### Application of the DNAzyme RFD-VV-M2 for *Vibrio vulnificus* Detection

Crab, fish, shrimp, and oysters were purchased from a local market and confirmed to be free of *V. vulnificus* by real-time PCR before use (Panicker et al., 2004). For the first step, in plastic boxes, the crab, fish, shrimp, and oysters were immersed in 1 L of seawater spiked with *V. vulnificus*. The final concentration of *V. vulnificus* was 10^7^ CFU/ml, and the boxes were kept at room temperature for 4 h. Then, 25 g of tissue samples were collected, homogenized, and centrifuged at 12,000 rpm for 5 min and filtered with a 0.22-μm disposable filter. Finally, 30 μl of the supernatant was taken for DNAzyme detection. For the second step, the shrimp were immersed in 1 L of seawater, spiked with desired amounts of *V. vulnificus*, and the final concentration of *V. vulnificus* was 10^7^ to 10^1^ CFU/ml. Then, the shrimp were kept in room for 4 h. Afterward, 25 g tissue of shrimp were collected, homogenized, centrifuged at 12,000 rpm for 5 min, and filtered with a 0.22-μm disposable filter. Finally, 30 μl was taken for DNAzyme detection.

### Real-Time Polymerase Chain Reaction

The *vvh* gene of *V. vulnificus* was detected using real-time PCR for comparison (Panicker et al., 2004). Briefly, the MonAmp^TM^ ChemoHS qPCR Mix (Monad Biotech, China) reaction mixture was prepared according to the manufacturer’s instructions. The qPCR cycling parameters were as follows: 94°C for 3 min, followed by 45 cycles of 94°C for 15 s, 56°C for 15 s, and 72°C for 25 s on a StepOne^TM^ real-time PCR System (Applied Biosystems, CA, United States).

## Results

### *In vitro* Selection

**FIGURE 3:**
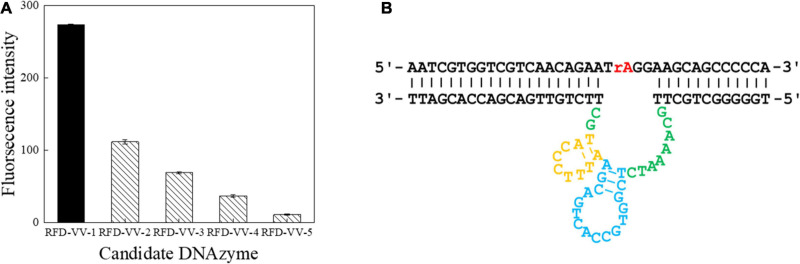
Detection of catalytic capabilities using the top five sequences. **(A)** The top five sequences were named RFD-VV-1 to RFD-VV-5. In the tests, 4 μl of sequences (5 μM) was used. The error bars represent the mean and standard error of three repeats. **(B)** Secondary structure of the RFD-VV-M1 DNAzyme.

A total of seven screening cycles were conducted, and negative selection was performed on rounds three and five to avoid non-specific division. The final PCR products were sequenced by Sangon Biotech. We received a total of 189,889 sequences from which the obtained candidate sequence was sorted. We chose the top five sequences, which are summarized in Table 2. Besides, the top 50 sequences are listed in Supplementary Table 1. FAM tag substrate and DNAzymes were used to form complexes, and fluorescence was detected once they were mixed with CEM-VV. The top five sequences were detected. Finally, detection results indicated RFD-VV-1 had the best catalytic capability (Figure 3A). The IDT Oligoanalyzer 3.1 software^1^ was then used to analyze the secondary structure of the first sequence in Figure 3B (RFD-VV-1), which has a conserved stem-loop structure.

**TABLE 2:** High throughput sequencing.

**No.**	**Aligned sequence (5′-3′)**	**%**
1	———GCAAAATCTCGGTGCCACTGACGAATTTCCCATGC———–	5.17
2	———CTTCTAGTCCTATTCACGACACCCCCCCGCGGTATC———–	2.71
3	———GTTTACCCCTGCAGCGAGAAGCGTGGTCACGCAC ———–	1.34
4	———CATGGTCCTATTGACTGCTCCAATGTAACCCGGCC————	1.00
5	———CATGGTCCTATTGACTGCTCCAATGTAACCCGGCC————	0.37

### Sequencing and Truncated Sequence Analysis of DNAzyme

**FIGURE 4:**
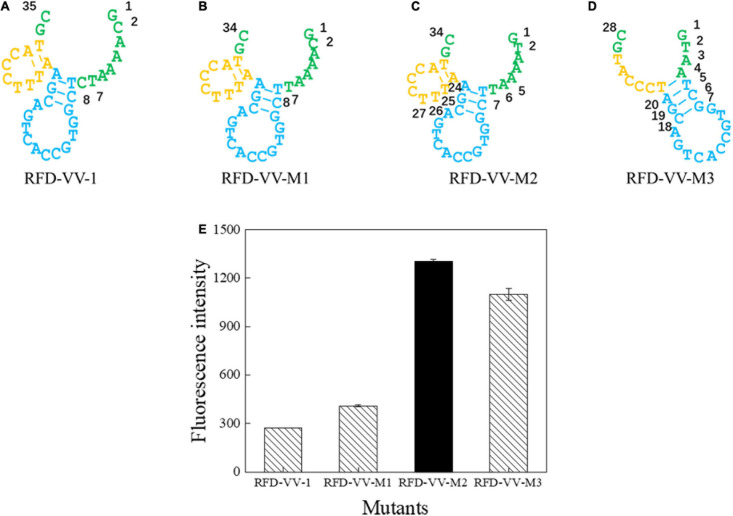
Catalytic core sequences of **(A)** RFD-VV-1, **(B)** RFD-VV-M1, **(C)** RFD-VV-M2, and **(D)** RFD-VV-M3. **(E)** Activity of mutant DNAzymes. The error bars represent the mean and standard error of three repeats.

Next, we sought to truncate the sequences to verify their activity. First, we removed the eighth base of the RFD-VV-1 (Figure 4A) core region; the resulting sequence will hereinafter be referred to as RFD-VV-M1 (Figure 4B). Additionally, we replaced the second base of RFD-VV-M1 with base T; the resulting sequence will hereinafter be referred to as RFD-VV-M2 (Figure 4C). Last, we removed bases 5–7 and bases 24–26 of RFD-VV-M2, thus, forming a new long hairpin structure with two loops; the resulting sequence will hereinafter be referred to as RFD-VV-M3 (Figure 4D).

We detected the activity of the truncated DNAzymes according to the description of enzyme assays. Finally, all results are shown in Figure 4E. RFD-VV-M2 exhibited the highest activity and will be used for subsequent experiments.

### Optimization of Reaction Conditions

**FIGURE 5:**
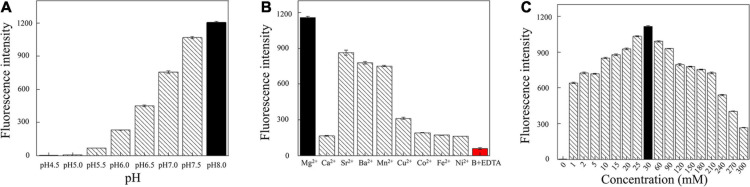
**(A)** Cleavage at different pH values. **(B)** Effect of different divalent metal ions on the cleavage activity. Adding 300 mM EDTA fully inhibited cleavage (all DNAzyme complexes were formed in buffer B). **(C)** Effect of different Mg^2+^ concentrations on cleavage activity.

Next, we optimized the reaction conditions for DNAzyme RFD-VV-M2. As shown in Figure 5A, the activity was highest at a pH of 8.0. Moreover, given that DNAzyme activity requires the presence of bivalent metal ions, divalent metal ion concentrations were optimized. As shown in Figure 5B, under the pH 8.0, DNAzymes have the highest activity in the presence of Mg^2+^. Concretely, DNAzyme RFD-VV-M2 performance was optimal when the Mg^2+^ concentration was 30 mM (Figure 5C). Finally, the optimized buffer (buffer B; 100 mM HEPES, 300 mM NaCl, 30 mM MgCl_2_ 0.02%, Tween 20, pH 8.0) was kept for subsequent experiments.

### Specificity and Sensitivity Detection

**FIGURE 6:**
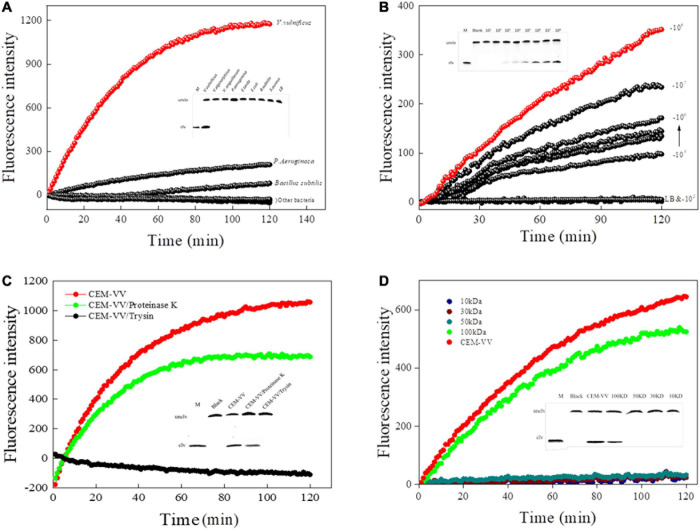
**(A)** The biosensor-based assay responded to RFD-VV-M2 in a blank culture medium and seven bacteria. Inset: Cleaving gel image for RFD-VV-M2 specificity detection within 1 h. **(B)** Biosensor assay of RFD-VV-M2 using CEM-VV from different concentrations of *V. vulnificus*. Inset: Gel micrograph showing the activity for 1 h. **(C)** The RFD-VV-M2-based biosensor exhibited no signal in response to trypsin-treated CEM-VV. Inset: Gel micrograph showing the biosensor activity. **(D)** Assessment of the molecular weight of the target protein. Inset: Gel images of the response to different molecular weight samples; all the cleavage reactions were conducted in buffer B for 1 h.

The specificity of RFD-VV-M2 toward different bacterial CEM was then characterized under optimal reaction conditions (buffer B; 100 mM HEPES, 300 mM NaCl, 30 mM MgCl_2_ 0.02%, Tween 20, pH 8.0). Results indicated that only *V. vulnificus* elicited a reaction from the RFD-VV-M2 sensor. Therefore, DNAzyme RFD-VV-M2 had high specificity for *V. vulnificus* (Figure 6A). Furthermore, the results of the 15% dPAGE confirmed the specificity. The sensor also remained specific to *V. vulnificus* compared with other bacteria.

Regarding the sensitivity of RFD-VV-M2, fluorescence increased with higher CEM-VV concentration. As the concentrations of *V. vulnificus* CEM-VV increased from 2.2 × 10^3^ to 2.2 × 10^8^ CFU/ml, the detection limit reached 2.2 × 10^3^ CFU/ml (Figure 6B).

### A Protein-Dependent DNAzyme Sensitivity

To identify the target of DNAzyme RFD-VV-M2, the CEM-VV was treated with protease K and trypsin. As shown in Figure 6C, the CEM-VV treated with trypsin could not induce the reaction. In contrast, protease K did not affect the reaction between CEM-VV and RFD-VV-M2. Therefore, these results suggest that the target was a protein that could be digested by trypsin. Furthermore, the molecular weight of the target protein was estimated between 50 and 100 kDa (Figure 6D). The results of electrophoresis also confirmed the results of fluorescence.

### *Vibrio vulnificus* Detection in Aquatic Products

**FIGURE 7:**
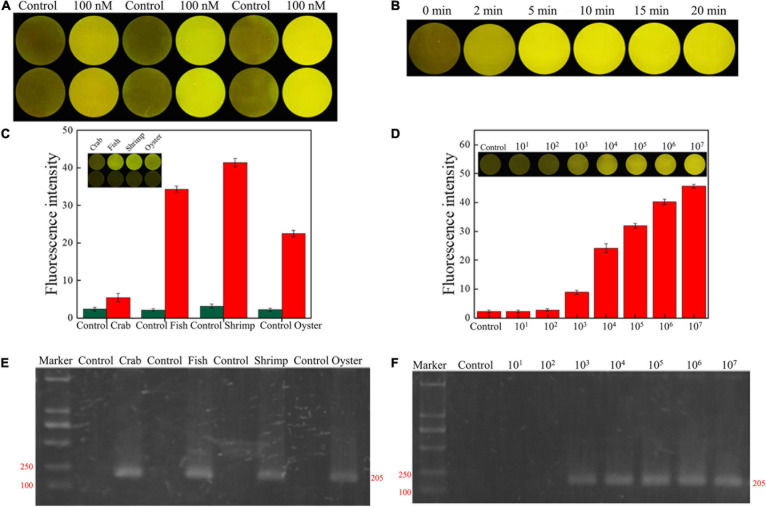
**(A)** RFD-VV-M2 concentration optimization (100–300 mM) on the sensor. **(B)** Optimization of fluorescence reaction time (0–20 min). **(C)** Detection of *V. vulnificus* in crab, fish, shrimp, and oyster samples under the concentration of 10^7^ CFU/ml. Red columns indicated different detection. Control: negative control. Inset: An active sensor exhibiting a positive fluorescent signal. **(D)** Detection of different concentrations of *V. vulnificus* in infected shrimp. Inset: The tissue fluid of infected shrimp rendered a fluorescent signal within 5 min. Control: negative control. **(E)** Corresponding agarose gel electrophoresis results of real-time PCR for *V. vulnificus* detection with primers L-vvh and R-vvh. M, D2000 Plus DNA marker; Control, negative control. **(F)** Corresponding agarose gel electrophoresis results of real-time PCR for shrimps spiked with *V. vulnificus.* M, D2000 Plus DNA marker; Control: negative control.

The concentration and reaction time of RFD-VV-M2 in the sensor was optimized to make a significant contrast between the background fluorescence intensity and the detection fluorescence intensity. When the concentration of DNAzyme was 300 nm, the fluorescence signal intensity generated by detection was the highest in Figure 7A. Finally, CEM-VV was then added to the prepared sensor, and when the reaction was taken at 5 min, a clear and obvious phenomenon of fluorescence signal was generated, and the fluorescence signal became clearer with time in Figure 7B.

Seafood contaminated with *V. vulnificus* (crab, fish, shrimp, and oysters) were detected with the DNAzyme RFD-VV-M2 sensor and the qPCR method for comparison. A robust fluorescence signal could be distinguished after 5 min of adding samples (Figures 7C,E). This indicated that the RFD-VV-M2-based sensor could rapidly and effectively detect *V. vulnificus* in contaminated seafood. Moreover, shrimp infected with 10^3^ CFU/ml *V. vulnificus* also produced clear fluorescent signals (Figure 7D). Importantly, the detection results of the RFD-VV-M2 sensor were consistent with the qPCR results as shown in Figure 7F and Tables 3, 4.

**TABLE 3:** Detection of *V. vulnificus* in spiked seafood samples.

**Seafood sample**	**Spike concentration (CFU/mL)**	***Vibrio vulnificus* (CICC 21615)**	
		**DNAzyme**	**Real-time PCR**
Negative control	0	−	22.25 ± 0.20
Positive control	10^7^	+	8.82 ± 0.06
Crab	10^7^	+	8.69 ± 0.05
Fish	10^7^	+	9.34 ± 0.06
Shrimp	10^7^	+	8.99 ± 0.08
Oyster	10^7^	+	8.67 ± 0.04

*+, positive result; −, negative result.*

**TABLE 4:** Detection of *V. vulnificus* in spiked seafood samples.

**Seafood sample**	**Spike concentration (CFU/mL)**	***Vibrio vulnificus* (CICC 21615)**	
		** DNAzyme**	**Real-time PCR (*Ct*)**
Shrimp	Control	−	22.25 ± 0.20^*a*^
	10^1^	−	22.14 ± 0.14^*a*^
	10^2^	−	22.10 ± 0.21^*a*^
	10^3^	+	19.47 ± 0.10^*b*^
	10^4^	+	17.33 ± 0.29^*c*^
	10^5^	+	14.82 ± 0.24^*d*^
	10^6^	+	12.16 ± 0.33^*e*^
	10^7^	+	8.87 ± 0.18^*f*^

*+, positive result; −, negative result; different letters represent significant differences (*P* < 0.05) between different spike concentrations.*

## Discussion

Seafood safety management has become an increasing challenge due to higher demands and logistical issues (Lincoln, 2019). Therefore, rapid and convenient detection methods for pathogenic bacteria in aquatic products are in high demand. DNAzyme-based biosensors have good potential for field detection of aquatic pathogens because of their agility, specificity, sensitivity, and minimal dependence on complex equipment (Ali et al., 2017). Therefore, DNAzyme-based detection methods have been developed for many common pathogens. *V. vulnificus* is among the most important pathogenic bacteria in seafood, and therefore, a detection method for this pathogen based on DNAzyme would be an excellent solution for rapid on-site detection.

At present, the detection of *V. vulnificus* is mainly performed by traditional culture methods (Hartnell et al., 2018), such as plate counting and the MPN method, both of which entail complex processes such as overnight culture, selective plate separation, biochemical identification, and serological experiments. These procedures are not only time consuming and labor intensive but are also prone to contamination with other bacteria in the sample, which may interfere with the identification of target bacteria.

In recent years, molecular biology techniques such as polymerase chain reaction (PCR) (Han and Ge, 2010) and quantitative PCR (qPCR) (Surasilp et al., 2011) have been widely used in the quantitative detection of *V. vulnificus*. Compared with traditional culture methods, real-time PCR is fast, simple, and sensitive. However, the experimental environment and the skills of the technician directly affect the amplification efficiency of the reaction and the quality of the standard curve, thus, affecting the detection accuracy. LAMP (loop-mediated isothermal amplification) (Wang et al., 2016) technology can be used for field detection, but it is greatly affected by the environment, which may easily lead to false-positive results. Moreover, compared with recombinase polymerase amplification (RPA) (Yang et al., 2020), LAMP has higher protein activity and higher reagent cost.

Compared with the above-described methods, the DNAzyme RFD-VV-M2 exhibited a high specificity toward *V. vulnificus*, and its limit of detection was also very low (10^3^ CFU/ml). Moreover, the biosensor showed good performance in detecting *V. vulnificus*-contaminated aquatic products. The design of the sensor provides a rapid and simple method for bacterial detection and provides a new and effective means for medical diagnosis. More importantly, the implementation of such sensors would be simple and low cost.

## Conclusion

A DNAzyme library was successfully screened, and a DNAzyme-based biosensor for rapid *V. vulnificus* detection was developed and optimized based on the selected DNAzyme candidate. The results of *V. vulnificus* detection could be observed within 5 to 10 min. Moreover, the DNAzyme RFD-VV-M2 sensor had a detection limit as low as 2.2 × 10^3^ CFU/ml. Therefore, the proposed method can effectively detect *V. vulnificus* in aquatic products and provides a framework for the development of other rapid detection methods for pathogenic bacteria, thus, contributing to the development of aquaculture and the reduction of human health risks.

## Data Availability Statement

The original contributions presented in the study are included in the article/Supplementary Material, further inquiries can be directed to the corresponding authors.

## Author Contributions

ML and SW designed the research. SF, CM, XPT, XM, MQ, HW, XQT, and JL conducted the research. SF, ML, and SW wrote the manuscript. SF and CM analyzed the data. ML directed the project. All authors contributed to the article and approved the submitted version.

## Conflict of Interest

The authors declare that the research was conducted in the absence of any commercial or financial relationships that could be construed as a potential conflict of interest.

## Footnotes

1 https://sg.idtdna.com/UNAFold
