# Supplementary material for: Detection of Vibrio vulnificus in Seafood With a DNAzyme-Based Biosensor
Source: Front Microbiol. 2021 Jun 4;12:655845. doi: 10.3389/fmicb.2021.655845 (PMC8213197; doi:10.3389/fmicb.2021.655845)
Supplement: Supplementary file 1 [file Table_1.docx]

**Detection of** ***Vibrio vulnificus* in seafood with a DNAzyme-based biosensor**

Shihui Fan^a,b,c^, Chao Ma^a,b,c^, Xiaopeng Tian^a,b,c^, Xiaoyi Ma ^a,b,c^, Mingcan Qin^a,b,c^, Hangjie Wu^a,b,c^, Xueqing Tian^a,b,c^, Jing Lu ^a,b,c^, Mingsheng Lyu ^a,b,c,∗^, Shujun Wang^a,b,c∗^

^a^Jiangsu Key Laboratory of Marine Bioresources and Environment/Jiangsu Key Laboratory of Marine Biotechnology, Jiangsu Ocean University, Lianyungang 222005, China

^b^Co-Innovation Center of Jiangsu Marine Bio-industry Technology, Jiangsu Ocean University, Lianyungang 222005, China

^c^Jiangsu Marine Resources Development Research Institute, Lianyungang, 222005, PR China

**Table S1** High throughput sequencing of top 50

| **No.** | **Sequence** | **Enrichment rate (%)** |
| --- | --- | --- |
| 1 | GCAAAATCTCGGTGCCACTGACGAATTTCCCATGCAAGCAG | 0.0517 |
| 2 | CTTCTAGTCCTATTCACGACACCCCCCCGCGGTATCAAGG | 0.0271 |
| 3 | GTTTACCCCTGCAGCGAGAAGCGTGGTCACGCACAAGCAG | 0.0135 |
| 4 | CATGGTCCTATTGACTGCTCCAATGTAACCCGGCCAAGCAG | 0.0100 |
| 5 | GCAATAACTGCTGGCCACGGACGATTATCACTGCCAAGCAG | 0.0037 |
| 6 | CTTTAAGTTCTGCGCCATCACCCCCGCGCGACCTCAAGCAG | 0.0024 |
| 7 | GTTGACCCCGACAGCGACAAGGCTGCTCGCTCCAAAGCAG | 0.0020 |
| 8 | GGTTGACCCCCTACAGGACGGCGTCATGCTGATACAAGCAG | 0.0016 |
| 9 | GCACCGGTTAAAACCACGAACGCCGGCCCACATGCAAGCAG | 0.0015 |
| 10 | GTTGTCCCCTGCAGCTGGGAAGGTTTCACCACGTAAAGCAG | 0.0015 |
| 11 | GGACTAGCCTCCTGTGACGAGTCAATGGCCCGACCAAGCAG | 0.0011 |
| 12 | GGACTTCTATTGCCTGGCGCTTCCCGAGCCGGTCCAAGCAG | 0.0011 |
| 13 | GCAGCGACCTGACCCCCTACTTTGCCTCAGGGGCTAAGCG | 0.0009 |
| 14 | TTACATTCTTGTCCGCCACCCCTCCGCGACCTTCAAGCAG | 0.0009 |
| 15 | CTTAGGGTCCTGCTGACTTCGTTCCAGGGCGATCCAAGCAG | 0.0004 |
| 16 | GTTGACCACTTACCCCGGCAGAGGGACTTCCCTTTAAGCAG | 0.0004 |
| 17 | TGTTGACCCTGCGCAGCACGTGACCCCCTCTCTGCAAGCAG | 0.0003 |
| 18 | CACTGTCGTCTTCCACCGCCTATCCTCACCCTCTCAAGCAG | 0.0003 |
| 19 | TGTTGACCCCATGAGCCGGACACTTTCCCTCCGAAAAGCAAG | 0.0003 |
| 20 | GCAGCGACCCATGACCCCTTATGTTCACCACGGTGAAGCAG | 0.0002 |
| 21 | GCCACTACGACATCCTCCCAAGGCTTGTCAACGACAAGCAG | 0.0002 |
| 22 | GCCACGACGACAGCTACCATCCTAGGCCGGGGCCAAGCAG | 0.0002 |
| 23 | GCCGTACTTCCGTCGCCTGCGTTCCATGGCGATCCAAGCAG | 0.0002 |
| 24 | CAAAATCTCGATGCCACTGACGAATTTCCCATGCAAGCAG | 0.0002 |
| 25 | CGCTGTACCGTTGACAGACACCCCCCCGCGACGTCAAGCAG | 0.0002 |
| 26 | CTTCTAGTCCTATTCACGACACCCCCCGCGGTATCAAGG | 0.0001 |
| 27 | GCAAAATCTCGGCGCCACTGACGAATTTCCCATGCAAGCAG | 0.0001 |
| 28 | GCCTCTGACCATCCTCTAGCCGGTGACGACAGCTCCAG | 0.0001 |
| 29 | CGCGTAGCGTCTTTGACGCGCGATGATGCCCGCCCAAGCAG | 0.0001 |
| 30 | GCACGCTACCTGATGCCGCTCAATGTGGCCCGACCAAGCAG | 0.0001 |
| 31 | GTTTACCCCTGCAGCGAGAAGCGTGGTCACGCACAGGCAG | 0.0001 |
| 32 | GCAAAATCTCGGTGCCACTGACGAATTTCCCATGCAGGCAG | 0.0001 |
| 33 | GCAAAATCCCGGTGCCACTGACGAATTTCCCATGCAAGCAG | 0.0001 |
| 34 | CGTTGAGACCCTTCCGGGGCGTTGCGATCGATCCAAGCAG | 0.0001 |
| 35 | CCTCTAGTCCTATTCACGACACCCCCCCGCGGTATCAAGG | 0.0001 |
| 36 | GTTGACCCCTACAGCGGCGGGCGCTAAAGCCTACAAGCAG | 0.0001 |
| 37 | GCAAAATCTCGGTGCCACTGACGAATTTCCCATGCGAGCAG | 0.0001 |
| 38 | GCAAAATCTCGGTGCCACTGACGAATTTCCCACGCAAGCAG | 0.0001 |
| 39 | CGATGCGGTGACGACTTGGGTTCACGTTGCGTTCCAAGCAG | 0.0001 |
| 40 | GCAAAATCTCGGTGCCACTGGCGAATTTCCCATGCAAGCAG | 0.0001 |
| 41 | GCTCCGGGGGACCGCGACGTGACCGCTACTGCTTTAAGCAG | 0.0000 |
| 42 | ATGTTGACCCCGACAGCGAAGGGCCAGGCTCCTAAAAGCAG | 0.0000 |
| 43 | GCAAAGTCTCGGTGCCACTGACGAATTTCCCATGCAAGCAG | 0.0000 |
| 44 | GCAAAATCTTGGTGCCACTGACGAATTTCCCATGCAAGCAG | 0.0000 |
| 45 | GCAAAACCTCGGTGCCACTGACGAATTTCCCATGCAAGCAG | 0.0000 |
| 46 | GCAGCGACCATGACCCCACCCTGTTCGCACAAGGCAAGCAG | 0.0000 |
| 47 | AGCAAAATCTCGGTGCCACTGACGAATTTCCCATGCAAGCAG | 0.0000 |
| 48 | GCCACTACGACAGCCCGGCCCGCATGACTACCGCCAAGCAG | 0.0000 |
| 49 | GCAAAATCTCAGTGCCACTGACGAATTTCCCATGCAAGCAG | 0.0000 |
| 50 | CTTCTAGTCCTATTCACGGCACCCCCCCGCGGTATCAAGG | 0.0000 |
